# Daily Maximum Temperatures Induce Lagged Effects on Leaf Unfolding in Temperate Woody Species Across Large Elevational Gradients

**DOI:** 10.3389/fpls.2019.00398

**Published:** 2019-03-28

**Authors:** Christof Bigler, Yann Vitasse

**Affiliations:** ^1^Forest Ecology, Institute of Terrestrial Ecosystems, Department of Environmental Systems Science, ETH Zürich, Zurich, Switzerland; ^2^SwissForestLab, Birmensdorf, Switzerland; ^3^Disturbance Ecology, Forest Dynamics, Swiss Federal Research Institute WSL, Birmensdorf, Switzerland

**Keywords:** phenology, conifers, broadleaved species, maximum temperature, distributed lag models, lag effects, multivariate meta-analysis, elevation

## Abstract

The timing of leaf unfolding in temperate woody species is predominantly controlled by the seasonal course of temperature in late winter and early spring. However, quantifying lagged temperature effects on spring phenology is still challenging. Here, we aimed at investigating lagged and potentially non-linear effects of daily maximum temperatures on the probability of leaf unfolding in temperate woody species growing across large elevational gradients. We analyzed 5280 observations of leaf-out time of four tree species (European beech, horse chestnut, European larch, Norway spruce) and one shrub species (common hazel) that were recorded by volunteers over 40 years at 42 locations in Switzerland. We used a case-crossover sampling design to match leaf-out dates with control dates (i.e., dates before or after leaf-out), and analyzed these data with conditional logistic regression accounting for lagged temperature effects over 60 days. Multivariate meta-analyses were used to synthesize lagged temperature and elevational effects on leaf unfolding across multiple phenological stations. Temperature effects on the probability of leaf unfolding were largest at relatively short lags (i.e., within ca. 10 days) and decreased with increasing lags. Short- to mid-term effects (i.e., within ca. 10 to 20 days) were larger for late-leafing species known to be photoperiod-sensitive (beech, Norway spruce). Temperature effects increased for the broadleaved species (horse chestnut, hazel, beech) with decreasing elevation, particularly within ca. 10 to 40 days, i.e., leaf unfolding occurs more rapidly at low elevations for a given daily maximum temperature. Our novel findings provide evidence of cumulative and long-term temperature effects on leaf unfolding, whereby the efficiency of relatively high temperatures to trigger leaf-out becomes higher shortly before bud burst. These lagged associations between temperature and leaf unfolding improve our understanding of phenological responses across temperate woody species with differing ecological requirements that occur along elevational gradients.

## Introduction

In temperate ecosystems, recurring biological phenomena are predominantly controlled by the cyclic, seasonal course of weather conditions (Forrest and Miller-Rushing, 2010; Schwartz, 2013). For example, temperature is a key environmental driver of the timing of trees’ spring phenophases such as leaf unfolding or flowering (Chuine, 2000; Polgar and Primack, 2011), with photoperiod playing an additional role for some species (Basler and Körner, 2012; Way and Montgomery, 2015). The strong dependency of phenological processes on temperature is further reflected in the phenological shift of trees’ leaf-out time along elevational gradients where temperature varies strongly over short distances (Vitasse et al., 2009; Bigler and Bugmann, 2018; Vitasse et al., 2018).

Leaf-out is typically observed when relatively warm spring temperatures prevail for a certain time. While one or a few warm days in late winter or early spring do not immediately result in leaf unfolding, a series of warm days in late spring may lead to premature leaf-out. Thus, the processes that ultimately induce leaf unfolding have already started several weeks or months before. Prior to leaf-out, vegetative buds of temperate woody species undergo a period of dormancy during which visible growth of buds is temporary suspended (Lang et al., 1987). Bud dormancy has been commonly divided into three physiological phases (Lang et al., 1987; Horvath et al., 2003): First, *paradormancy* from summer to early fall, which is imposed by distal hormonal control from outside the buds but within the plant (Cline and Deppong, 1999). Second, *endodormancy* from early fall to mid-winter, which is induced by chilling temperatures and shorter days and further regulated by physiological mechanisms occurring within the buds (Horvath et al., 2003). Endodormancy is released by exposure to cool temperatures in mid-winter (“chilling” temperatures between ca. 0 to 10°C; Polgar and Primack, 2011; Hänninen, 2016), and for some species photoperiod may also play a role (Caffarra et al., 2011a,b). Third, unfavorable environmental conditions (e.g., low temperatures, short days or drought) during *ecodormancy* prevent the buds from leafing out, until warm temperatures (“forcing” temperatures above ca. 5°C; Chuine, 2000; Polgar and Primack, 2011) and increasing day length in early spring induce bud burst and leaf unfolding. Chilling and forcing temperatures are assumed to be negatively related, i.e., a longer period of chilling requires a shorter period of forcing until bud break occurs (Murray et al., 1989; Laube et al., 2014). A longer photoperiod further increases the sensitivity of buds to forcing temperatures for some species such as European beech (Basler and Körner, 2014). Formulating and testing hypotheses about potential temperature effects on leaf unfolding remains challenging due to (1) the contrasting effects of chilling and forcing on bud development (Cannell and Smith, 1983; Murray et al., 1989), (2) the loosely defined ranges of chilling and forcing temperatures (Chuine, 2000; Luedeling et al., 2013; Delpierre et al., 2016), (3) the gradual transitions between or the overlap of the three dormancy phases (Cooke et al., 2012), (4) the relative importance of photoperiod versus temperature (Chuine et al., 2010; Körner and Basler, 2010; Vitasse and Basler, 2013), and (5) the correlation between temperature and photoperiod due to the similar seasonal course.

Because weather conditions in spring can drastically change from year to year (Körner and Basler, 2010; Vitasse et al., 2014b), dates of leaf unfolding may differ by one or even 2 months at the same site (Sparks and Carey, 1995; Lenz et al., 2016). Leaf unfolding is further known to be delayed at higher elevations (Vitasse et al., 2018), where it occurs after less accumulated warmth compared to lower elevations (Güsewell et al., 2017). To improve our understanding of leaf phenological processes, we would actually require to link continuous measurements of the development, growth or physiological activity of vegetative buds from bud set in summer to bud burst in spring with high-resolution weather data (Cooke et al., 2012). However, molecular or physiological changes that would reflect transient phases during bud dormancy remain to be found. Furthermore, long-term measurements of leaf phenology are typically restricted to observed dates of bud burst or leaf unfolding only. Thus, relating these phenological events to the driving forces such as lagged temperature effects is notoriously challenging.

A large range of statistical phenology models have been developed to predict the leaf-out time of forest trees in response to weather variability (Hänninen, 2016). The simplest statistical approach uses a linear model to relate the time of leaf unfolding (e.g., day of year) to some aggregated measure of temperature (e.g., growing degree days, mean temperature in late winter and spring, or monthly temperature variables; Sparks and Carey, 1995; Menzel et al., 2006; Ibáñez et al., 2010; Roberts, 2012). A drawback of this approach is that the period needs to be fixed over which monthly or seasonal weather variables are considered. Because the leaf-out time varies in response to the year-to-year changes of temperature and along elevation (Güsewell et al., 2017), fixed periods are likely to increase uncertainty in these models or may induce artifacts, e.g., if weather conditions following mean leaf unfolding dates are considered that turn out to have significant influences in specific years only. Unlike these linear models that include relatively coarse weather variables as predictors, more complex statistical approaches have been devised that rely on daily weather data such as (i) partial least squares regression (Roberts, 2008; Luedeling and Gassner, 2012); (ii) penalized signal regression (Roberts, 2008; Hudson, 2010; Roberts et al., 2015); or (iii) hierarchical state-space models (Clark et al., 2014a,b). Although these statistical models consider temperature data prior to leaf unfolding, they do not explicitly model lagged temperature effects, which may yet improve our understanding of how temperature regulates leaf unfolding (Hudson, 2010).

In addition to statistical phenology models, many process-based models have been proposed to predict spring phenology based on the hypothetical processes occurring during the endo- and ecodormancy (Chuine et al., 2013; Basler, 2016; Hänninen, 2016). These models may be classified as whether they account for (i) forcing temperature in spring only; (ii) chilling temperature during winter and forcing temperature in spring; or (iii) additionally photoperiod as an interacting factor during both the endodormancy and ecodormancy phases. These process-based models do not explicitly account for lagged daily weather effects, but rather integrate daily or hourly temperatures over a defined period. Besides, simpler process-based models often perform as well as or even outperform more complex models that require more parameters and are therefore often overparameterized, reflecting the poor understanding of the processes regulating spring phenology (Linkosalo et al., 2008; Vitasse et al., 2011; Clark et al., 2014b; Olsson and Jönsson, 2014; Basler, 2016).

The goal of this study was to investigate lagged and potentially non-linear effects of daily maximum temperature on the probability of leaf unfolding in temperate woody species along large elevational gradients. Although most of the phenological models have used daily mean temperature, here we used maximum temperature, because (i) daytime temperature seems to exert stronger effects on leaf unfolding than nighttime temperature (Fu et al., 2016) and (ii) we expect stronger lag effects of temperature beyond specific thresholds. The investigated tree and shrub species included early- to late-leafing species as well as photoperiod-sensitive and -insensitive species. We addressed the following research questions:

(1) For how long do daily maximum temperatures of varying intensity affect leaf unfolding? We expect longer-term effects of cooler temperatures due to lower development rates of buds, but more immediate effects of warmer temperatures.
(2) Do species differ with respect to the lagged associations between temperature and leaf unfolding? We expect stronger effects of temperature in late-leafing species, because leaf-out tends to occur at higher temperatures.
(3) Does the lagged association between temperature and leaf unfolding change along elevation? We expect stronger effects of higher temperatures at lower elevations, as chilling requirements to break endodormancy may not always be fulfilled due to the warmer conditions.

## Materials and Methods

### Phenological Data

We used phenological observations of five tree and shrub species that were available from the phenological network of MeteoSwiss, the Swiss Federal Office of Meteorology and Climatology (Defila and Clot, 2001). We considered both broadleaved species (European beech, *Fagus sylvatica* L.; horse chestnut, *Aesculus hippocastanum* L.; common hazel, *Corylus avellana* L.) and conifer species (European larch, *Larix decidua* Mill.; Norway spruce, *Picea abies* (L.) H. Karst). The set of species ranges from early-leafing species with high frost resistance during leaf unfolding (larch, horse chestnut, hazel), to beech as an intermediate-leafing species with intermediate frost resistance and Norway spruce as a late-leafing species with low frost resistance (Lenz et al., 2013; Vitasse et al., 2014a; Bigler and Bugmann, 2018). While larch, horse chestnut and hazel rely mainly on temperature as a trigger of leaf unfolding, beech and Norway spruce rely on both temperature and photoperiod (Heide, 1993; Basler and Körner, 2012).

We included only phenological series without missing observations of leaf unfolding from 1972 to 2011 (i.e., 40 years of data; Supplementary Table S1 and Supplementary Figure S1). Phenological observations were conducted weekly by one voluntary observer per station, applying the same protocol for phenology monitoring across stations. The date of leaf unfolding was recorded when approximately 50% of the leaves of one or several trees or shrubs were unfolded, i.e., the leaf surface and leaf base is visible in broadleaved species, or the young needle bundles start to open and spread in conifer species. The dataset included 5280 observed dates of leaf unfolding from 42 phenological stations (beech: 26 stations; hazel: 23 stations; horse chestnut: 29 stations; larch: 35 stations; Norway spruce: 19 stations; Supplementary Table S1). The data had been checked for plausibility and consistency in a previous study (Bigler and Bugmann, 2018). All five species were observed jointly at five stations, four species at fourteen stations, three species at nine stations, two species at ten stations, and at four stations only one species was observed (Supplementary Table S1). The stations were distributed across Switzerland covering a perimeter of 12896 km^2^ and an elevational range from 200 to 1800 m a.s.l. (beech: 200 to 1240 m a.s.l.; hazel: 350 to 1120 m a.s.l.; horse chestnut: 200 to 1120 m a.s.l.; larch: 200 to 1800 m a.s.l.; Norway spruce: 200 to 1800 m a.s.l.; Supplementary Table S1). The climate regimes included mean annual temperature ranges from 2.9 to 12.4°C, and annual precipitation ranges from 751 to 1960 mm.

### Weather Data

We used spatially interpolated daily temperature data from 1 January 1972 to 31 December 2011 (i.e., 14610 values) at each of the 42 phenological stations. We selected daily maximum temperature T_max_ as predictor variable (Supplementary Figure S1), because leaf unfolding responds more to daytime temperature than to nighttime temperature (Hanes, 2014; Piao et al., 2015; Fu et al., 2016). However, because daily mean temperature generally provides more accurate phenological predictions when using process-based models, we have also tested daily mean temperature (T_mean_) as predictor variable to check the consistency of the resulting patterns. The daily temperature data were available for a grid of 100 m resolution across Switzerland, and were derived using the DAYMET interpolation algorithm (Thornton et al., 1997) applied to measured weather data from MeteoSwiss climate stations and a digital elevation model. The interpolated temperature data were provided by the Landscape Dynamics group at the Swiss Federal Research Institute WSL (Birmensdorf, Switzerland). For each phenological station, we averaged T_max_ (T_mean_) of the nearest grid cell and the surrounding eight neighboring cells to get robust estimates of T_max_ (T_mean_). Although the locations of the observed trees and shrubs may differ from the coordinates of the phenological stations by several hundreds of meters or in some cases even by several kilometers, we assumed that the interpolated temperature data still reflect relatively accurately the weather conditions of the trees and shrubs.

### Data Analysis

**FIGURE 1:**
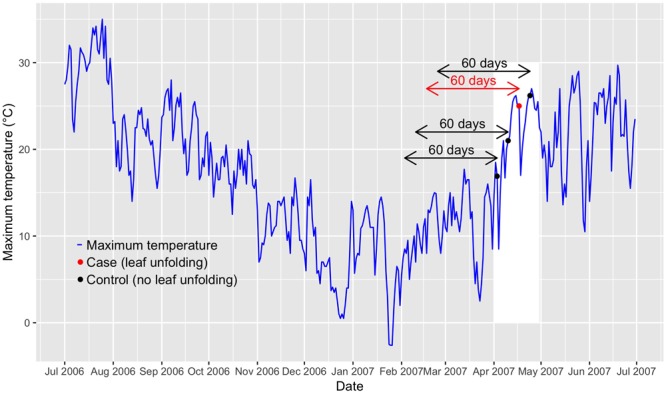
Visualization of the time-stratified case-crossover design. The example shows the observed leaf-out time of beech at the phenological station Liestal (350 m a.s.l.; Supplementary Table S1) in Switzerland and T_max_ (maximum temperature) from July 2006 until end of June 2007. The red dot indicates the case (date of leaf unfolding: 17 April 2007), the black dots indicate the controls (dates before or after leaf unfolding: 3 April 2007, 10 April 2007, and 24 April 2007). The arrows indicate the 60-day lags that are considered in the distributed lag models. April 2007 is delimited by a white shaded box.

**FIGURE 2:**
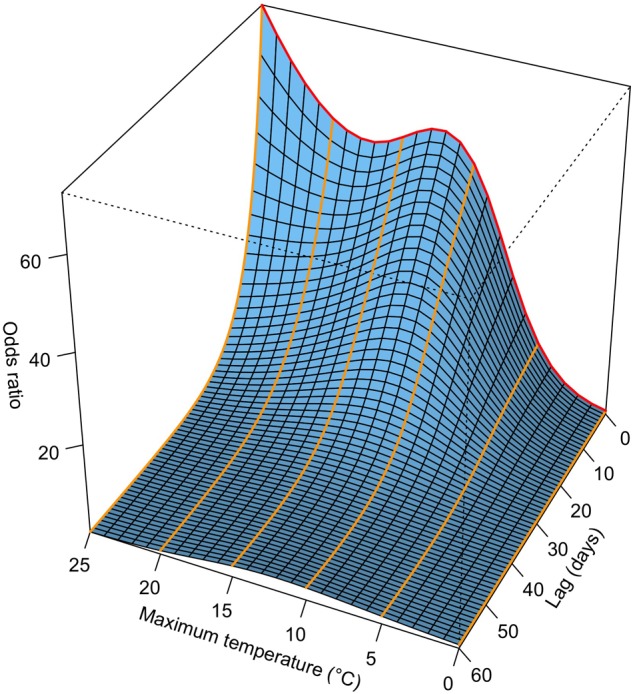
Visualization of a DLNM (distributed lag non-linear model). The example shows the odds ratio of beech at the phenological station Liestal (350 m a.s.l.; Supplementary Table S1) in Switzerland along T_max_ (maximum temperature) and lag dimension. A conditional logistic regression model was fitted to time-stratified case-crossover data (Figure 1) from 1972 to 2011. The odds ratios are interpreted as the ratio between the odds of a specific T_max_ at a specific lag compared to the odds of the reference T_max_ (0°C) at the same lag (eq. 3). The red line indicates the odds ratio along T_max_ at a lag of 0 days, the orange lines indicate the odds ratios along the lag dimension at T_max_ of 0°C (odds ratio = 1), 5°C, 10°C, 15°C, 20°C, and 25°C.

Rather than predicting leaf-out dates, we quantified the probability of leaf unfolding to occur under specific weather conditions. To quantify lagged temperature effects on leaf unfolding, we combined several statistical methods that are common in environmental epidemiology (Schwartz, 2000; Nitta et al., 2010; Gasparrini and Armstrong, 2013) but that have not been used in phenological studies (Hudson, 2010). We used the following approaches (see below for further details): (1) a case-crossover design to compare T_max_ during case days (i.e., dates when leaf unfolding was observed) with control days (i.e., dates before or after leaf unfolding), accounting for lagged temperature effects up to 60 days before each case and control (Figure 1); (2) a conditional logistic regression to predict for each species and phenological station the effect of T_max_ on the probability of leaf unfolding, which extends the logistic regression by accounting for stratified data (i.e., sets of cases and controls); and (3) multivariate meta-analyses to synthesize associations between T_max_ and leaf unfolding across multiple phenological stations. The same approaches were used to analyze the data also based on T_mean_, which provided qualitatively similar results as with T_max_ as predictor variable (see Supplementary Figures S2, S3).

#### Time-Stratified Case-Crossover Design

Case-crossover designs allow to compare environmental conditions during case days (i.e., dates when the phenological event occurred) that are matched to one or several control days (Maclure, 1991). This sampling design constitutes a self-matching of each phenological station and thus controls for time-invariant or only slowly time-varying confounders such as topography, vegetation etc. Several strategies have been proposed to match cases to controls. We selected the time-stratified case-crossover design (Lumley and Levy, 2000), i.e., each case (date of leaf unfolding) was matched to controls of the same year, month and weekday (Figure 1). Each set of 1 case and 3 to 4 controls is considered a stratum. The gaps of 6 days between case and control days (see Figure 1) reduce serial autocorrelation (Janes et al., 2005a). By restricting the controls to the same month as the case, only periods are considered when leaf unfolding of vegetative buds may actually be observed. The time-stratified case-crossover design further controls for potential bias due to seasonal and long-term changes in T_max_ (Janes et al., 2005a), which ensures unbiased parameter estimates in conditional logistic regression (see below). Sampling of control days only before rare or non-recurrent events such as leaf unfolding would result in biased parameter estimates (Janes et al., 2005b). The dataset across all species and the entire 40-year period included 5280 cases and 17931 controls (beech: 1040 cases, 3563 controls; hazel: 920 cases, 3113 controls; horse chestnut: 1160 cases, 3921 controls; larch: 1400 cases, 4726 controls; Norway spruce: 760 cases, 2608 controls).

#### Conditional Logistic Regression

Matched data from time-stratified case-crossover designs are commonly analyzed using conditional logistic regression (Breslow et al., 1978), which accounts for the non-independence of the stratified data. The probability of observing a case in stratum k (π_k_) may be defined as a stratum-specific logistic regression (Breslow et al., 1978; Hosmer et al., 2013):

(1) $$\begin{array}{c} \pi_{k}\left(y=1\;|\;x\right)=\frac{e^{\alpha_{k}+\beta x}}{1+e^{\alpha_{k}+\beta x}} \end{array}$$

where *y* is the dependent variable (*y* = 1: case, i.e., leaf unfolding occurs; *y* = 0: control, i.e., leaf unfolding does not occur), *x* is the predictor variable (e.g., T_max_ or a function of T_max_), α_k_ is a stratum-specific constant, and β is the coefficient for variable *x*, which is estimated across all strata. In this study, the strata *k* correspond to the years during the observation period (i.e., *k* = 1972, …, 2011). Equation 1 may be rewritten as log-odds:

(2) $$\begin{array}{c} log\frac{\pi_{k}\left(y=1\;|\;x\right)}{1-\pi_{k}\left(y=1\;|\;x\right)}=\alpha_{k}+\beta x \end{array}$$

Thus, a unit increase in variable *x* changes the log-odds by β. Equation 2 contains no intercept, but a stratum-specific constant α_k_, which cancels out in the conditional likelihood.

The effect of *x* on the probability of observing a case is typically expressed as odds ratio, i.e., the ratio between the odds of a value *x*_1_ and the odds of a reference value *x*_0_:

(3) $$\begin{array}{c} odds\;ratio=\;\frac{\pi_{k}\left(y=1\;|\;x_{1}\right)/\left(1-\;\pi_{k}\left(y=1\;|\;x_{1}\right)\right)}{\pi_{k}\left(y=1\;|\;x_{0}\right)/\left(1-\;\pi_{k}\left(y=1\;|\;x_{0}\right)\right)}=\;e^{\beta \left(x_{1}-x_{0}\right)} \end{array}$$

We used the statistical computing software R (R Core Team, 2018), version 3.5.1, to fit conditional logistic regression models (function “clogit” in the package “survival,” version 2.42-4). We selected an exact calculation method to maximize the conditional likelihood.

#### Distributed Lag Models

We expanded the conditional logistic regression (eq. 2) to account for lagged effects of T_max_ on leaf unfolding. In particular, we fitted distributed lag non-linear models (DLNMs) to the data, which allow for flexible and delayed associations between a response variable and a predictor variable along time (Gasparrini et al., 2010). The DLNM was defined as follows:

(4) $$\begin{array}{c} log\frac{\pi_{k}\left(y=1\;|\;x_{t},\;\ldots ,\;x_{t-l},\;\ldots ,\;x_{t-L}\right)}{1-\pi_{k}\left(y=1\;|\;x_{t},\;\ldots ,\;x_{t-l},\;\ldots ,\;x_{t-L}\right)}=\alpha_{k}+s\left(x_{t},\;\ldots ,\;x_{t-l},\;\ldots ,\;x_{t-L};\;\eta \right) \end{array}$$

where the smoothing function s(…) was represented by a bi-dimensional natural spline for the predictor variable *x*_t_ (here T_max_) at day t and lag days l (l = 0, …, L) and a vector of coefficients ***η*** (Gasparrini et al., 2010). We used natural cubic spline functions with two interior knots for both the predictor variable T_max_ and the lag dimension. T_max_ was restricted to the interval [0°C, 25°C] to prevent artifacts that may have occurred at particularly warm or cool stations due to extrapolations of temperature beyond the range of observed values. The maximum lag L was set at 60 days (Figure 1) based on findings in previous studies (Fu et al., 2015; Vitasse et al., 2018). The interior knots were selected to evenly split the range of T_max_ (knots at 8.3 and 16.7°C) and lag dimension (knots at 20 and 40 days) into three intervals. We calculated predictor-specific summaries at T_max_ of 5, 10, 15, and 20°C. Odds ratios (eq. 3) were calculated with respect to the reference value 0°C. Thus, we predicted odds ratios for lags from 0 to 60 days with T_max_ set to 5, 10, 15 and 20°C. We refer to Figure 2 for an illustrative example. Because odds ratios not only depend on the estimated model coefficients but also on the difference between T_max_ and the reference value of 0°C (eqs. 3 and 4), comparisons of the odds ratios are only valid across species, lags and elevations, but not across different values of T_max_. However, significance of the odds ratios may be compared at different values of T_max_. We used the R packages “dlnm” (Gasparrini, 2011), version 2.3.4, and “splines,” version 3.5.1, to calculate the DLNMs.

#### Multivariate Meta-Analyses

A multivariate meta-analysis combines estimates of several parameters (e.g., coefficients from a regression model) across models from several sites, which allows to estimate average parameters. We used multivariate meta-analyses to synthesize lagged effects of T_max_ on leaf unfolding across multiple phenological stations (Jackson et al., 2011; Gasparrini et al., 2012). Multivariate meta-analysis allows to (i) pool estimates of multi-parameter associations and quantify between-station variability, and (ii) consider meta-variables that vary across stations. Based on the estimated coefficients ^ η _i_ (eq. 4) and the corresponding variance-covariance matrix ^ S _i_ from each phenological station i, random-effects multivariate meta-analyses were conducted (Gasparrini and Armstrong, 2013). We further included elevation as a meta-variable in random-effects multivariate meta-regression models (Gasparrini and Armstrong, 2013). The models were fitted to the data using restricted maximum likelihood from the R package “mvmeta,” version 0.4.11 (Gasparrini et al., 2012).

## Results

**FIGURE 3:**
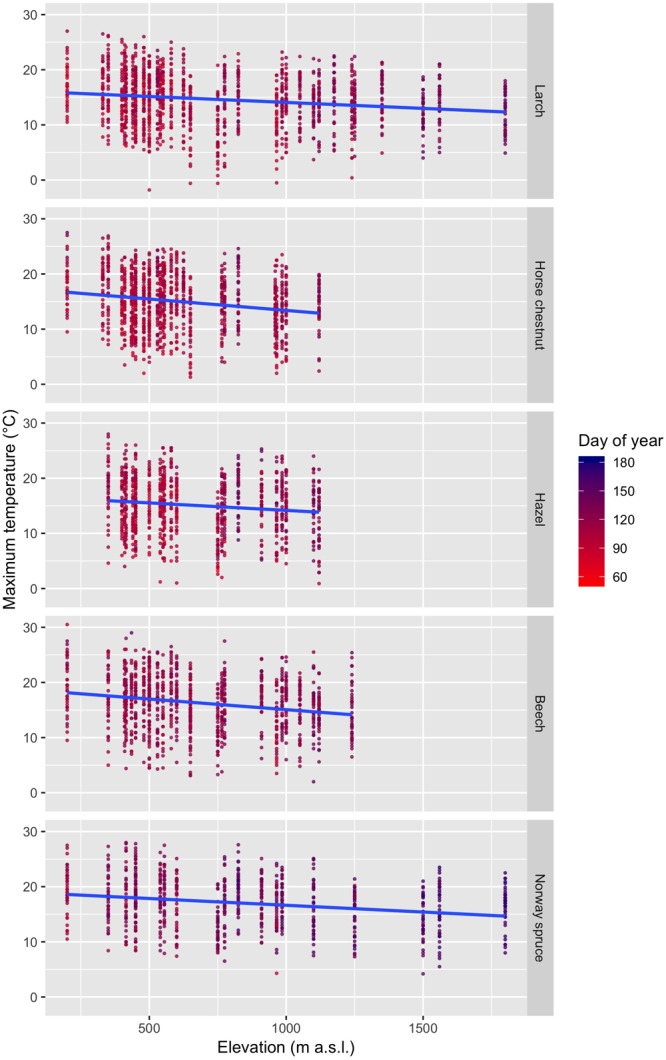
Change of T_max_ (maximum temperature) during the day of leaf unfolding with increasing elevation for larch, horse chestnut, hazel, beech, and Norway spruce. The species are ordered from early-leafing (top) to late-leafing (bottom). The blue lines indicate the regression lines based on the linear models.

Leaf unfolding was observed when relatively high daily maximum temperatures (T_max_) prevailed, which increased from early- to late-leafing species (Figure 3 and Supplementary Figure S1): larch, 14.6 ± 4.7°C (mean ± standard deviation); horse chestnut, 15.0 ± 4.9°C; hazel, 15.1 ± 5.0°C; beech, 16.3 ± 4.9°C; Norway spruce, 17.0 ± 4.6°C (species ordered from early- to late-leafing; Bigler and Bugmann, 2018). However, in some cases, leaf unfolding was recorded at low temperatures with *T*_max_ ≤ 5°C (Figure 3): larch, 2.43% of all observations; horse chestnut, 2.50%; hazel, 2.50%; beech, 1.63%; Norway spruce, 0.26%. The expected T_max_ during the day of observed leaf unfolding decreased with increasing elevation for all species (Figure 3): larch, -0.22°C/100 m; horse chestnut, -0.41°C/100 m; hazel, -0.27°C/100 m; beech, -0.38°C/100 m; Norway spruce, -0.25°C/100 m. Because the distribution of daily T_max_ during the day of leaf unfolding does not provide a proper account of temperature effects on leaf unfolding, we need to investigate the temperature profiles before leaf unfolding, which will be considered in the following analyses.

**FIGURE 4:**
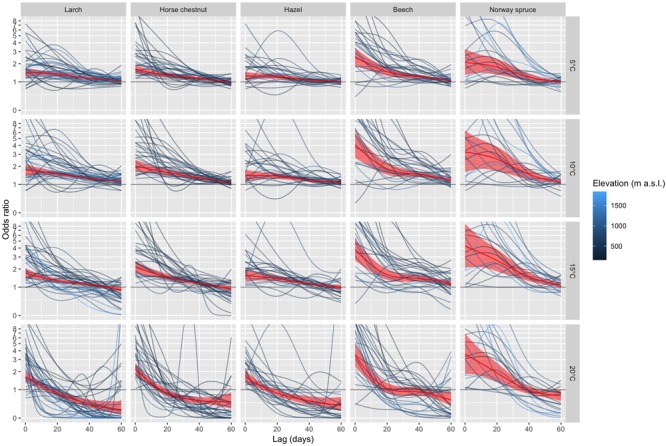
Summaries of DLNMs (distributed lag non-linear models) based on conditional logistic regression for larch, horse chestnut, hazel, beech, and Norway spruce. The odds ratios along the lag dimension are shown for T_max_ of 5°C, 10°C, 15°C, and 20°C. The species are ordered from early- to late-leafing species (left to right). The station-specific odds ratios are represented by the dark-blue lines (low-elevation stations) to light-blue lines (high-elevation stations). For sake of clarity, no confidence intervals are shown for the station-specific odds ratios. The red lines are the pooled estimates of the odds ratios (including 95% confidence intervals) from the multivariate meta-analysis. An inverse hyperbolic sine transformation has been applied to the *y*-axis to increase the visibility of smaller values.

For each species and station, we fitted a conditional logistic regression model with distributed lags (eq. 4) to the time-stratified data over the entire 40-year period (see Figure 2 for an example). The species-specific summaries of the odds ratios (eq. 3) for specific values of T_max_ (5, 10, 15, and 20°C) along the lag dimension are shown in Figure 4. At relatively short lags (0 to ca. 10 days), the odds ratios of most phenological stations were larger than 1, which tended to increase with decreasing elevation for some species (e.g., beech). The odds ratios of many phenological stations decreased with increasing lag (Figure 4), thus, the short-term (0 to ca. 10 days) effects of *T*_max_ ≥ 5°C on the probability of leaf unfolding were higher compared to the reference T_max_ of 0°C than the longer-term effects. At longer lags (ca. 50 to 60 days), the odds ratios were distributed around 1 when T_max_ was 5 or 10°C. Particularly for warmer temperatures (T_max_ of 15 and 20°C), the odds ratios dropped below 1 at longer lags for quite many stations, i.e., the probability of observing leaf unfolding 50 to 60 days following a warm day was lower than at 0°C. Such effects may occur, when leaf unfolding has already occurred (Figure 1 and Supplementary Figure S1). Overall, there was a relatively large variability among stations regarding the lagged association between T_max_ and leaf unfolding (Figure 4).

Based on the multivariate meta-analyses, the pooled estimates and 95% confidence intervals of the odds ratios provide a general overview on the change of the odds ratios and their significance with increasing lags (Figure 4). The early-leafing species (larch, horse chestnut, hazel) showed quite a similar pattern, i.e., short- (0 to ca. 10 days) to long-term effects (ca. 50 to 60 days) with significant odds ratios >1 were detected at T_max_ of 5 and 10°C, whereas short-term effects dominated at 20°C. For the later-leafing species beech and Norway spruce, the pooled odds ratios at short lags (0 to ca. 10 days) were larger than for the early-leafing species, but also suggest a higher variability among stations as indicated by the relatively wide confidence intervals (Figure 4). Larger odds ratios translate into more rapid leaf unfolding under identical temperatures. For example, the pooled odds ratio at T_max_ of 15°C and at a lag of 0 days (Figure 4) increased from hazel (1.6), larch (1.7), horse chestnut (2.1), beech (3.8) to Norway spruce (4.2), i.e., leaf unfolding of beech and Norway spruce occurs more rapidly than for hazel, larch and horse chestnut at this temperature. The pooled estimates based on the multivariate meta-analyses using T_mean_ resulted in qualitatively similar results (Supplementary Figure S2), though the odds ratios at short lags (0 to ca. 10 days) were a bit lower than for T_max_.

**FIGURE 5:**
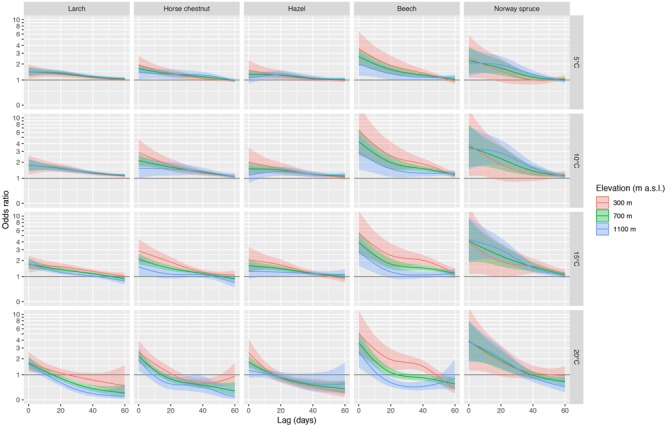
Summaries of DLNMs (distributed lag non-linear models) based on conditional logistic regression for larch, horse chestnut, hazel, beech, and Norway spruce. The odds ratios along the lag dimension are shown for T_max_ of 5°C, 10°C, 15°C, and 20°C. The species are ordered from early- to late-leafing species (left to right). The odds ratios for three elevations (300 m, 700 m, 1100 m a.s.l.) and 95% confidence intervals were estimated from the multivariate meta-regression. An inverse hyperbolic sine transformation has been applied to the *y*-axis to increase the visibility of smaller values.

The pooled odds ratios based on the multivariate meta-regression models showed distinct and significant effects along elevation (Figure 5). Particularly the broadleaved species (horse chestnut, hazel, beech) showed consistent elevational effects within ca. 10 to 60 days, i.e., odds ratios generally decreased from low elevation (300 m a.s.l.) to mid elevation (700 m a.s.l.) and high elevation (1100 m a.s.l.), which translates into more rapid leaf unfolding at lower elevations under identical temperatures. For example, the pooled odds ratio for beech at T_max_ of 15°C and at a lag of 0 days is 5.5 at 300 m a.s.l. and 2.8 at 1100 m a.s.l. (Figure 5), i.e., leaf unfolding of beech occurs more rapidly at lower elevations than at higher elevations at this temperature. The pooled estimates based on the multivariate meta-regressions using T_mean_ also resulted in similar results (Supplementary Figure S3), but with slightly lower odds ratios at short lags and generally wider 95% confidence intervals compared to T_max_.

## Discussion

Long-term series of observed leaf-out timings from conifers and broadleaved species provide evidence of species- and elevation-specific responses to temperature. The time-stratified case-crossover design combined with the statistical framework proved to be a suitable approach to (i) describe simultaneously the non-linear and delayed dependencies of leaf unfolding on daily temperature, and (ii) synthesize lagged associations between temperature and leaf unfolding across multiple phenological stations. We detected a decline in the lagged effects of varying maximum temperature (T_max_) on leaf unfolding. Thus, the strongest effects of T_max_ on the development of vegetative buds are expected within a few days, while lagged effects after ca. 30 days tend to fade out. Beech and Norway spruce, which are both sensitive to photoperiod and tend to leaf-out late, showed stronger short-term effects of T_max_ on leaf unfolding than larch, horse chestnut and hazel. Broadleaved species (horse chestnut, hazel, beech) showed a stronger short-term response to T_max_ at low elevations than at higher elevations.

### Lagged Effects of Temperature on Leaf Unfolding

We established lagged and non-linear effects of T_max_ on the probability of leaf unfolding. Temperature effects decrease with increasing lags as demonstrated by the pooled odds ratios of the multivariate meta-analyses (Figure 4). Thus, immediate temperature effects within ca. 10 days are more likely to induce leaf unfolding than lagged effects after ca. 30 days or more. Lower T_max_ (5 and 10°C) tends to stimulate leaf unfolding even at longer lags (i.e., after more than ca. 30 days). In contrast at T_max_ of 20°C this is the case only at relatively short lags and after more than ca. 10 days even turns into a lower odds compared to the reference T_max_ of 0°C. The latter effect is known as “harvesting” (Gasparrini et al., 2010). In other words, odds ratios decrease for events such as leaf-out dates after the event has occurred, because they occur only once per year.

Despite these relatively distinct effects of pooled odds ratios, we observed quite a high variability of lagged associations between T_max_ and leaf unfolding among phenological stations (Figure 4). Several reasons are likely to account for this heterogeneity: (1) Environmental conditions at the phenological stations do not only differ in terms of T_max_, but also with respect to further influences such as precipitation, radiation, topography or species composition, which may affect microclimate and site conditions as perceived by the trees. (2) The observed trees of the phenological network differ in terms of tree height, diameter, age, genetic status, social position within the forest stand, and the surrounding forest structure (e.g., forest edge vs. interior stand). For example ontogenetic differences between adult trees and seedlings may substantially affect the timing of leaf unfolding in broadleaved trees (Vitasse, 2013). (3) The true positions of the observed trees may differ from the coordinates of the phenological stations, i.e., the interpolated weather data do not fully represent the conditions experienced by the trees. (4) There might be an observer bias, because phenological observers may slightly differ in their assessment of the timing of leaf unfolding (Bison et al., 2018; Güsewell et al., 2018). Overall, however, there seems to be a pattern of lagged and non-linear associations between T_max_ and leaf unfolding that is common across stations.

The preseason, which is related to the concept of lagged temperature effects on leaf unfolding, is defined as the period before leaf-out time when specific weather conditions are associated with leaf unfolding (Güsewell et al., 2017). The length of the preseason has been estimated in several studies during which temperatures exert a statistically significant influence on the timing of leaf unfolding. Correlation coefficients and partial correlation coefficients, respectively, between mean dates of leaf unfolding and temperatures averaged over varying periods before leaf-out have been used to determine the length of the preseason. Fu et al. (2015) found for seven broadleaved tree species across Europe, including horse chestnut and beech among other species, optimal preseason lengths of 45 to 60 days based on daily T_mean_. However, there was substantial within-species variability across sites ranging from 15 days to 4 months. The preseason for 24 plant species across Europe was estimated based on monthly T_max_ to range between 0 and 3 months for most combinations of species, sites and years (Piao et al., 2015). Using data from the phenological network of MeteoSwiss, Güsewell et al. (2017) estimated the optimal preseason to range between less than 60 days for beech and up to more than 70 days for Norway spruce, while Vitasse et al. (2018) found the highest correlations for the preseason to range from 40 to 50 days (beech) to ca. 65 days (Norway spruce). Thus, these estimates of preseason length are generally consistent with the findings in our study, even if methodologically quite different approaches were used. However, correlations between mean leaf-out time and mean temperature do not account for (i) variability of leaf-out time across sites or years; (ii) daily resolution of temperature data; and (iii) the time-ordered structure of the predictors (e.g., sequence of daily temperature).

Lagged temperature effects on leaf unfolding such as based on distributed lag models in our study or based on partial correlation coefficients (Fu et al., 2015; Piao et al., 2015; Güsewell et al., 2017) may be interpreted as indirect evidence of instantaneous thermal effects on the continuous development, growth or physiological activity of vegetative buds during dormancy and bud burst (Clark et al., 2014b). Increasing temperatures in spring induce deacclimation processes, whereby frost resistance decreases for example due to decreased levels of soluble carbohydrates, that finally result in bud burst (Pagter and Arora, 2013). Deacclimation occurs with some lag following exposure to warm temperatures, typically within a few days to weeks (Pagter and Arora, 2013). While it would be desirable to take continuous and more process-related measurements during dormancy and bud burst, observational and experimental studies are restricted in this regard. Assessing the state of dormancy in vegetative buds tends to be difficult, because molecular or physiological markers are lacking (Cooke et al., 2012) and because changes of bud morphology are hardly visible prior to bud break (but see Basler and Körner, 2014).

### Associations Between Temperature and Leaf Unfolding Differ Across Species

The lagged associations between temperature and leaf unfolding revealed differences between early-leafing, photoperiod-insensitive species (larch, horse chestnut, hazel) and late-leafing, photoperiod-sensitive species (beech, Norway spruce). Both beech and Norway spruce showed stronger short- to mid-term responses (i.e., within ca. 10 to 20 days) to any of the specified temperatures (Figure 4). Thus, leaf unfolding of beech and Norway spruce is expected to occur more rapidly than for larch, horse chestnut and hazel when periods with the same temperatures prevail. These patterns may reflect different leafing strategies to reduce frost risk following unseasonably warm spells, i.e., either by slow deacclimation in the early-leafing species or by more rapid deacclimation in the late-leafing species (Heide, 1974; Pagter and Arora, 2013).

Beech and Norway spruce are sensitive to photoperiod (Heide, 1974; Basler and Körner, 2012, 2014; Vitasse and Basler, 2013), and both species tend to leaf out rather late because of the lower frost resistance at leaf-out compared to the other species (Repo, 1992; Taschler et al., 2004; Lenz et al., 2013; Vitasse et al., 2014a; Bigler and Bugmann, 2018). Leaf unfolding of beech lags the early-leafing species in this study by ca. 1 to 2 weeks, Norway spruce by ca. 2 to 3 weeks or more (Bigler and Bugmann, 2018). Sensitivity to photoperiod or high chilling requirement to break dormancy may be considered as an additional safeguard allowing frost sensitive species to further minimize the risk of frost damage (Körner and Basler, 2010; Way and Montgomery, 2015), e.g., if a warm early spring or late winter is followed by a series of severe frost events. Species-specific rates of deacclimation in spring result in different development rates of vegetative buds (Pagter and Arora, 2013). Thus, when the photoperiodic and chilling requirements are fulfilled, the vegetative buds of the trees respond more rapidly to increasing temperatures (Körner and Basler, 2010).

The photoperiod-insensitive species and species having low chilling requirements (larch, horse chestnut, hazel), respectively, tend to leaf out rather early (Bigler and Bugmann, 2018). These species mainly rely on forcing temperatures as a trigger of bud burst and leaf unfolding (Basler and Körner, 2012). To compensate for the higher frost risk, they maintain a higher frost resistance during leaf-out. The weaker leaf phenological response to warmth compared to the late-leafing photoperiod-sensitive species may be interpreted as a further strategy to reduce the risk of frost damages (Lenz et al., 2016), because an overly rapid bud development may result in early leaf unfolding occurring simultaneously with or shortly after severe frost. Because the weather conditions may strongly change during early spring, and because there is a particularly high uncertainty regarding the occurrence of frost, plants have to take precautionary measures to reduce the risk of frost damages following early warm spells (Pagter and Arora, 2013). Advancing the timing of leaf unfolding by just 1–3 weeks strongly increases the risk of frost damages due to the shorter return intervals of frost events during the leaf-out period (Lenz et al., 2016).

Differences among tree species in terms of leaf-out times, frost resistance at the time of leaf-out and lagged temperature effects on leaf unfolding reflect varying strategies to cope with uncertain environmental conditions. Particularly late frost events in spring are considered ecologically and evolutionarily important events that may have severe consequences for the dynamics of tree populations, because physical and physiological damages occur that affect parts of trees or entire tree individuals (Inouye, 2000; Gu et al., 2008). Thus, frost is a strong selective pressure (Lenz et al., 2013) and is assumed to ultimately act as a key factor that determines tree species ranges (Chuine and Beaubien, 2001; Chuine, 2010; Körner et al., 2016).

### Effects of Elevation on the Association Between Temperature and Leaf Unfolding

The lagged association between temperature and leaf unfolding changes along elevation, particularly for broadleaved species (horse chestnut, hazel, beech), less so for conifers (larch, Norway spruce; Figure 5). The temperature effects increased for the broadleaved species with decreasing elevation, particularly within the first ca. 10 to 40 days, depending on species and specified temperature. Thus, under identical temperature conditions, leaf unfolding of these broadleaved species is expected to occur more rapidly at low elevation than at high elevation, assuming that chilling and photoperiod requirements are fulfilled. Some of these elevational effects may be explained with the decreasing T_max_ with increasing elevation (Figure 3 and Supplementary Figure S1), i.e., trees at higher elevations tend to leaf out at lower temperatures than trees at lower elevations.

The stronger response of horse chestnut, hazel and beech to temperature at lower elevations may indicate that a lack of chilling could be compensated by increased forcing temperatures. Beech as a photoperiod-sensitive species is further known to be more limited by daylength and chilling at lower elevations than at higher elevations (Vitasse and Basler, 2013). In this context, our findings may be interpreted that leaf unfolding of low-elevation beech trees responds strongly and relatively rapidly as soon as the photoperiodic and chilling requirements are fulfilled.

Leaf unfolding is delayed with increasing elevation as a consequence of the cooler temperatures (Lenz et al., 2016; Bigler and Bugmann, 2018; Vitasse et al., 2018). While temperatures generally decrease with increasing elevation, temperature variability tends to increase, thus uncertainty regarding the occurrence of frost is relatively high. Return intervals of frost events during the leaf-out period typically decrease with increasing elevation (Lenz et al., 2016), therefore, even relatively warm temperatures in spring or early summer at high elevation do not guarantee absence of frost. We interpret the lower odds ratios of the lagged association between temperature and leaf unfolding at higher elevations in our study as an adaption to reduce frost risk.

## Conclusion

The findings from our study provide evidence of lagged, non-linear temperature effects on leaf unfolding of temperate broadleaved species and conifers with contrasting leaf-out timing, frost resistance and photoperiodic requirements. Maximum temperatures induce relatively strong effects on leaf unfolding within a few days, while lagged effects over more than 1 month are weaker. Our findings demonstrate cumulative and long-term temperature effects on leaf unfolding, whereby relatively high temperatures before leaf unfolding accelerate bud development inducing rapid bud burst. We assume that such effects are not observed earlier in spring, because the buds are in a premature developmental state. Tree species respond differently to the impact of temperature: the immediate reaction of late-leafing, photoperiod-sensitive species is stronger, i.e., leaf unfolding occurs more rapidly than for early-leafing, photoperiod-insensitive species. Broadleaved species further show stronger temperature effects at low elevation than at high elevation, which translates into more rapid leaf unfolding with decreasing elevation under identical temperature conditions. The lagged associations between temperature and leaf unfolding improve our understanding on how the phenology of tree species with differing ecological requirements that occur along large elevational gradients respond to the cyclic, seasonal course of weather.

## Data Availability

Publicly available datasets were analyzed in this study. This data can be found here: https://gate.meteoswiss.ch/idaweb.

## Author Contributions

CB conceived and designed the study, processed and analyzed the data, and wrote the draft manuscript. CB and YV discussed the results. YV contributed to the writing of the manuscript. Both authors contributed to manuscript revision, read and approved the submitted version.

## Conflict of Interest Statement

The authors declare that the research was conducted in the absence of any commercial or financial relationships that could be construed as a potential conflict of interest.
