# Supplementary material for: Daily Maximum Temperatures Induce Lagged Effects on Leaf Unfolding in Temperate Woody Species Across Large Elevational Gradients
Source: Front Plant Sci. 2019 Mar 28;10:398. doi: 10.3389/fpls.2019.00398 (PMC6447654; doi:10.3389/fpls.2019.00398)
Supplement: Supplementary file 1 [file Data_Sheet_1.pdf]

Supplementary Material

Supplementary Table S1. Description of the phenological stations and the available phenological data for each study species. For each station, the station code and station name as well as WGS84 coordinates (latitude, northing; longitude, easting) and elevation are shown. The X-letters indicate that leaf-out dates of the corresponding species were observed from 1972 to 2011.

Station code	Station name	Latitude (°)	Longitude (°)	Elevation (m a.s.l.)	Beech	Hazel	Horse chestnut	Larch	Norway spruce
ADB	Adelboden	46.492	7.5611	1350				X	
ANR	Andeer	46.601	9.4226	985	X	X	X	X	X
APL	Appenzell	47.332	9.4154	775	X	X	X	X	X
BOD	Bondo GR	46.338	9.5559	825		X	X	X	X
CAR	Cartigny	46.177	6.0141	400		X	X	X	
CHI	Changins	46.396	6.2294	435	X		X		
DAD	Davos-Dorf	46.809	9.8366	1560				X	X
DIH	Diessenhofen	47.690	8.7440	410	X	X	X	X	
DOM	Domat-Ems	46.834	9.4574	580	X	X	X	X	
EHT	Escholzmatt	46.899	7.9323	910	X	X			X
ELP	Elm	46.922	9.1727	1000	X	X	X	X	
ENB	Entlebuch	46.987	8.0689	765	X	X	X		
KAN	Kandersteg	46.499	7.6657	1175				X	
LBU	Laufenburg	47.561	8.0631	330			X	X	
LCN	Locarno	46.169	8.7982	200	X		X	X	X
LEY	Leysin	46.339	7.0230	1250				X	X
LEZ	Lenzerheide	46.716	9.5315	1500				X	X
LIN	Linthal	46.913	9.0008	650	X		X	X	
LIT	Liestal	47.481	7.7305	350	X	X	X	X	X

LPM	Les Ponts-de-Martel	47.003	6.7286	1120	X	X	X	X	
MEH	Merishausen	47.764	8.6124	540		X	X	X	X
MTI	Moutier	47.279	7.3858	530	X		X	X	
MUG	Murg	47.108	9.2174	500	X		X	X	
PSO	Prato-Sornico	46.396	8.6348	750	X	X		X	X
SCL	Scuol	46.798	10.2948	1240	X			X	
SCW	Schoenenwerd	47.365	7.9990	450		X	X	X	X
SEO	Seon	47.345	8.1665	550	X	X	X	X	
SEW	Seewis Dorf	46.965	9.6321	960			X		
SGS	Sargans II	47.051	9.4392	480	X		X	X	
SMO	St. Moritz	46.498	9.8397	1800				X	X
SNN	Sarnen	46.894	8.2522	500	X	X	X	X	
VES	Versoix	46.278	6.1672	440		X	X	X	
VIL	Villchern	47.471	8.1549	415	X	X		X	X
VSA	La Valsainte	46.649	7.1825	1050	X			X	
WAD	Waedenswil	47.223	8.6798	480			X	X	
WAT	Wattwil SG	47.290	9.1044	625			X	X	
WIB	Wiliberg	47.280	8.0273	650	X				
WIH	Wildhaus	47.205	9.3526	1100	X	X		X	X
WYA	Wyu	47.256	7.8085	450	X	X	X	X	X
ZHP	Zuerich-MeteoSchweiz	47.378	8.5658	555		X	X		X
ZIZ	Zizers	46.931	9.5662	600	X	X	X		X
ZWS	Zweisimmen	46.555	7.3734	965	X	X	X	X	X

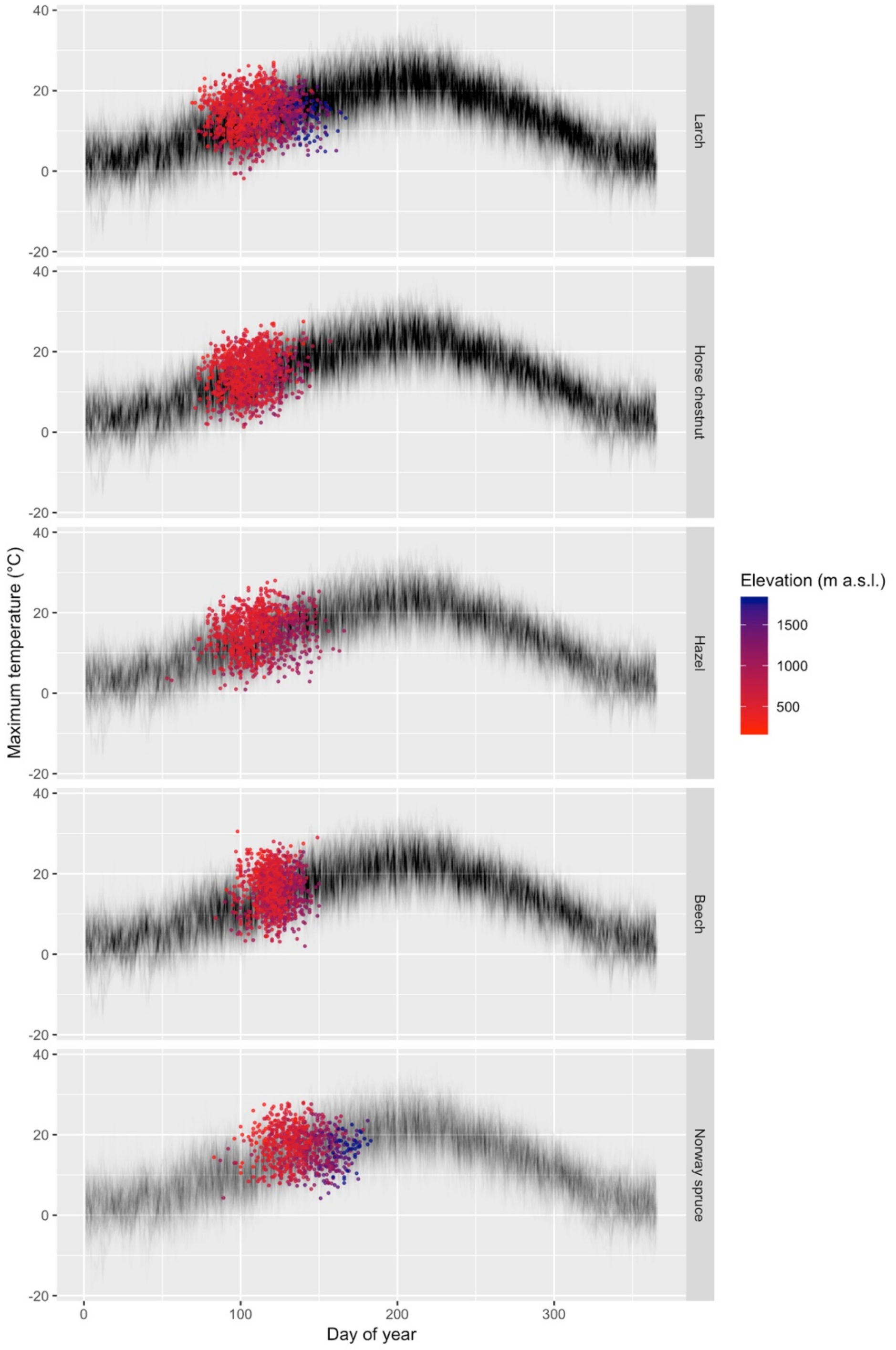

Supplementary Figure S1. Seasonal change in T_{max} (daily maximum temperature) for larch, horse chestnut, hazel, beech, and Norway spruce (thin black lines). Each line represents T_{max} from one year (1972 to 2011) at one station. The dots indicate the observed dates of leaf unfolding. The color gradient represents the elevation of the phenological stations (high-elevation stations: dark blue; low-elevation stations: red). The species are ordered from early- to late-leafing species (top to bottom).

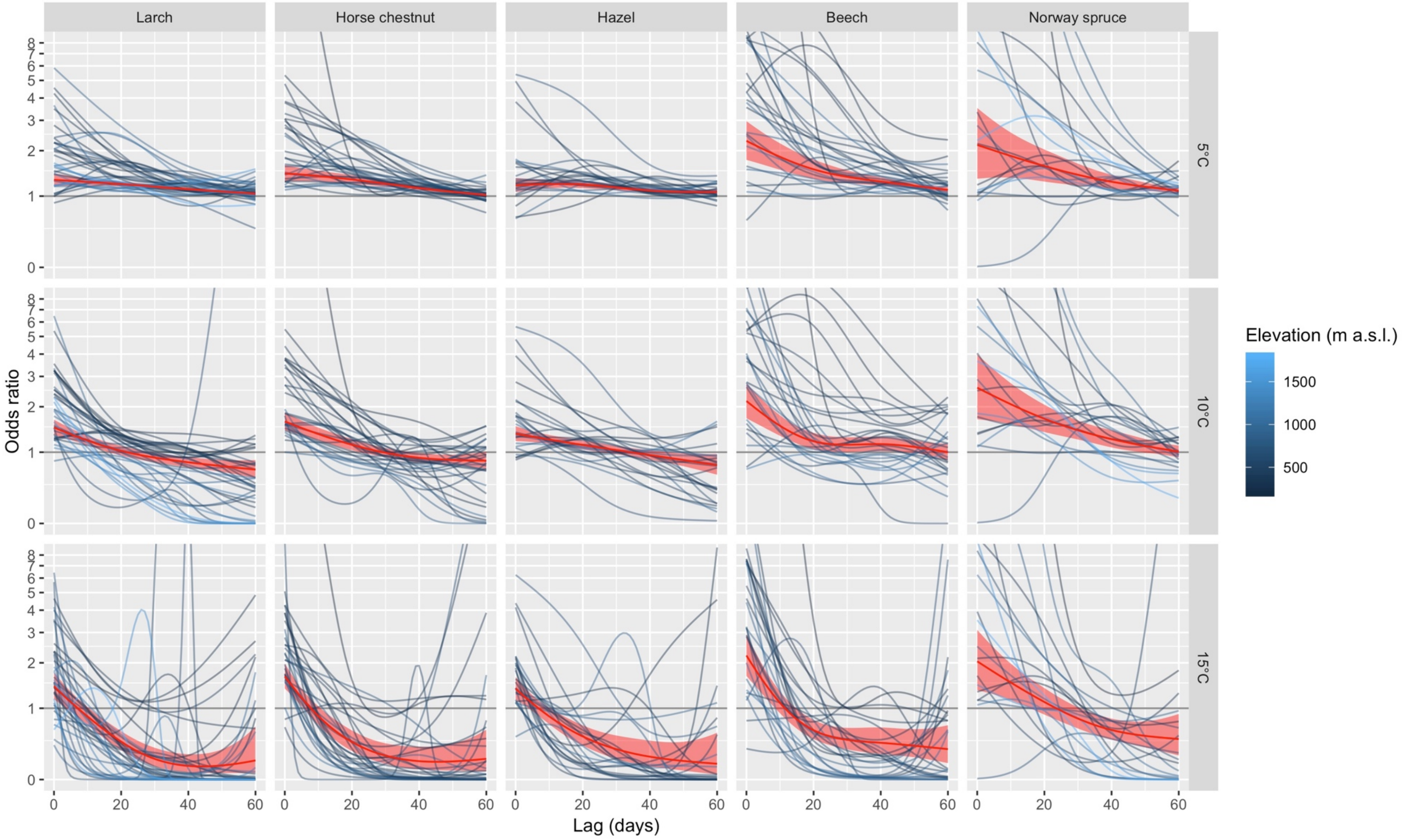

Supplementary Figure S2. Summaries of DLNMs (distributed lag non-linear models) based on conditional logistic regression for larch, horse chestnut, hazel, beech and Norway spruce. The odds ratios along the lag dimension are shown for T_{mean} of 5°C, 10°C, and 15°C. Because of lower T_{mean} compared to T_{max} (Fig. 4) odds ratios are not shown for 20°C. For further details see Fig. 4.

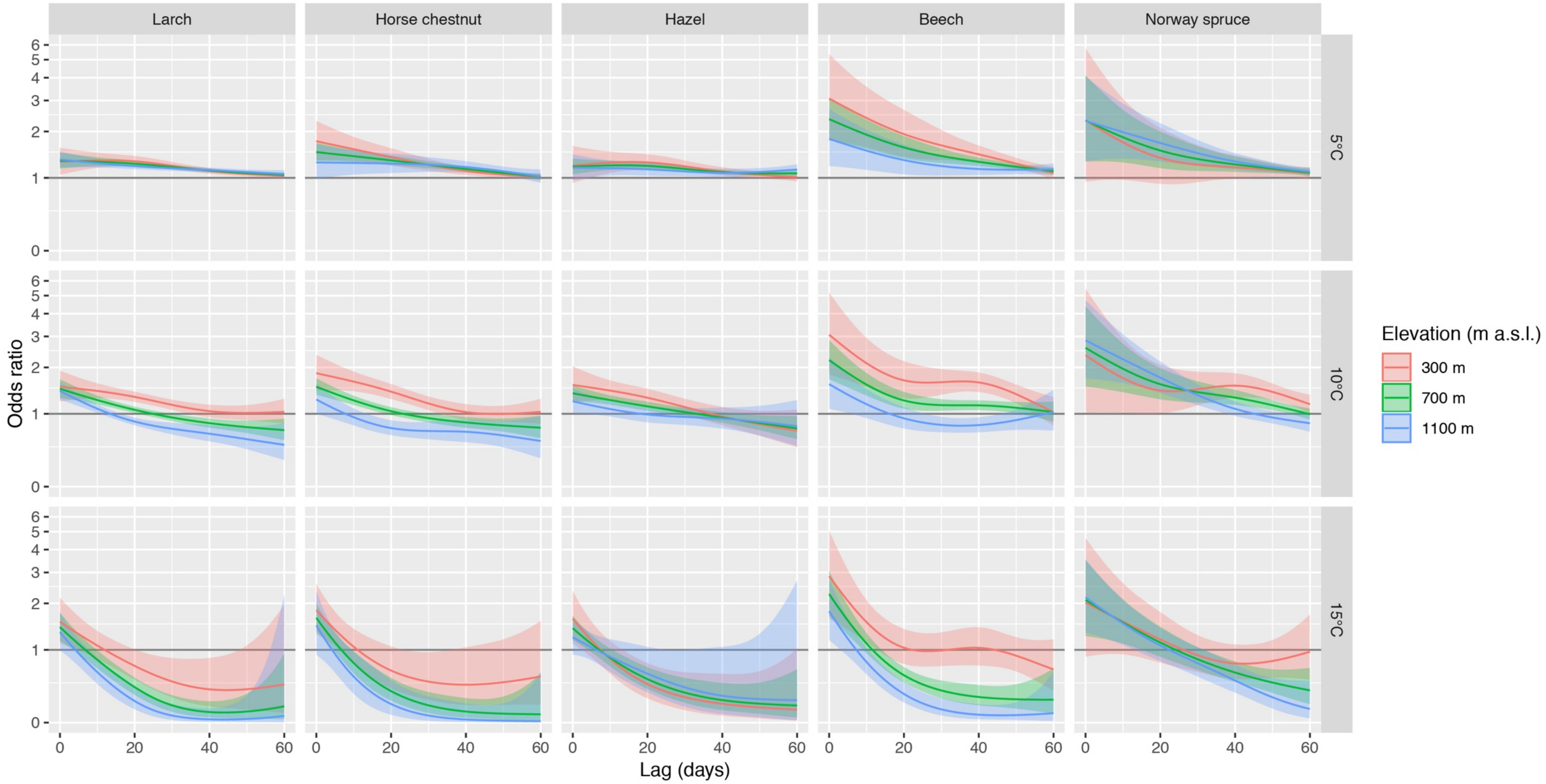

Supplementary Figure S3. Summaries of DLNMs (distributed lag non-linear models) based on conditional logistic regression for larch, horse chestnut, hazel, beech and Norway spruce. The odds ratios along the lag dimension are shown for T_{mean} of 5°C, 10°C, and 15°C. Because of lower T_{mean} compared to T_{max} (Fig. 5) odds ratios are not shown for 20°C. For further details see Fig. 5.
